# Good health information for people with a migration background

**DOI:** 10.1093/eurpub/ckac131.503

**Published:** 2022-10-25

**Authors:** S Ecker, M Weigl, S Gaiswinkler

**Affiliations:** Health, Society and Equity, Austrian National Public Health Institute, Vienna, Austria

## Abstract

**Background:**

Studies show that people with a migration background are often uncertain concerning the kinds of health services that are offered and where to turn to with which health concerns. Some factors, like fears or obstacles (e.g. deductibles), lead to the health system not being used (on time). This is particularly true for those who are socioeconomically or socially disadvantaged. The aim of the project was to get an insight into the information needs of this population group as a basis for producing and disseminating good health information in the future.

**Methods:**

In this qualitative study co-researchers conducted guided focus groups (separated by sex) or interviews in their first languages. In total, more than 100 people from 16 different countries of origin were involved in the survey.

**Results:**

In general, a substantial need for multilingual health information prepared in easy-to-understand language was expressed. Some health topics (e.g. the Austrian health system, mental health and available support) were mentioned by many participants. Other issues were relevant for few groups only (e.g. TCM). Gender differences can be seen in some groups but not in all. Besides acquaintances/relatives, general practitioners were named as main source of information. Indications can be derived on how health information should be prepared, designed and distributed. Besides multilingual health information, translation services are needed during appointments but also for the medical reports.

**Conclusions:**

When searching for health information, but also when trying to understand it, language barriers are a major obstacle. Multilingual and culture-sensitive explainer videos on selected topics are an appropriate medium for reaching a broad group of people. Low-threshold multilingual regional contact points could provide an important contribution to health equity, as a guide in the health system, providing information and translation services, and setting health promotion offers.

**Key messages:**

• Language barriers represent a major obstacle for vulnerable population groups with migration background in all stages of the health care process.

• There is a substantial need for adequately prepared multilingual health information.

